# The Clinical Value of Oxymatrine in Preventing Lamivudine Induced YMDD Mutation: A Meta-Analysis

**DOI:** 10.1155/2015/971616

**Published:** 2015-10-05

**Authors:** Min He, Yu Wu, Mengmeng Wang, Wenwen Chen, Weian Yuan, Jian Jiang

**Affiliations:** Department of Clinical Pharmacology, Shuguang Hospital Affiliated to Shanghai University of TCM, Shanghai 201203, China

## Abstract

Oxymatrine (OMTR) is widely used for the treatment of chronic hepatitis B (CHB) in China. Several reports revealed that combination of OMTR and lamivudine reduced the incidence of tyrosine- (Y-) methionine- (M-) aspartic acid- (D-) aspartic acid (D) (YMDD) mutations in CHB patients. The aim of this study was to evaluate the clinical value of oxymatrine in preventing lamivudine induced YMDD mutation using meta-analysis of data from published randomized controlled trials (RCTs) and to provide some useful information for clinical treatment and future research of YMDD mutation. The Cochrane Central Register of Controlled Trials, Medline, Science Citation Index, EMBASE, China National Knowledge Infrastructure, Wanfang Database, and China Biomedical Database were searched to identify RCTs that evaluated the incidence of YMDD-motif mutation to lamivudine therapy and lamivudine plus OMTR therapies in CHB patients. Data analysis was carried out with the use of RevMan 5.3.2. The literature search yielded 324 studies, and 16 RCTs matched the selection criteria. Overall, the incidence of YMDD mutation was significantly lower in patients treated with lamivudine plus OMTR than in patients treated with lamivudine alone (11.14% versus 28.18%; RR: 0.41; 95% CI: 0.33–0.52; *p* < 0.05). The exact outcome needs to perform rigorously designed, multicenter, and large randomized controlled trials.

## 1. Introduction

Chronic infection of hepatitis B virus (HBV) poses serious public health problems because of the high prevalence rates in many parts of the world and adverse long-term clinical outcomes, including premature deaths from hepatic decompensation, cirrhosis, and hepatocellular carcinoma [1]. The majority of countries in Asia have low-income economies and are at high endemicity of HBV infection [2]. It is estimated in China that there are 120 million chronically infected carriers; up to 12 million people suffer from chronic hepatitis B (CHB), and about 300,000 people die each year [3].

Currently nucleoside/nucleotide analogues (NAs) are the main available treatments for CHB. The approved NAs include lamivudine, telbivudine, adefovir, tenofovir, and entecavir [1]. However, treatment of chronic HBV infection is a complex task, and HBV drug resistance is one of the most significant factors in treatment failure for CHB [4]. Selective pressure from either the immune response or the use of NA in antiviral therapy could be driving the emergence of HBV drug-resistance mutants [4–6]. Drug-resistance mutations occur in the reverse transcriptase region of the HBV polymerase gene and spontaneously arise during viral replication. These mutations alter the hepatitis B surface (HBs) protein and in some cases reduce binding to HBs antibodies, as well as reducing susceptibility of HBV to NA. The spread of NA-resistant HBV may impact the efficacy of antiviral treatment and hepatitis B immunization programmers [5, 6]. The long-term benefits of antiviral treatment are limited by the resistance of HBV [4]. Moreover, since the number of HBV antiviral drugs is limited, transmission of mutant virus is of particular importance for HBV infection as mutations that confer cross-reactivity can leave patients with few therapy options.

The tyrosine-methionine-aspartate-aspartate (YMDD) is one of the most common HBV drug-resistance mutations, which is in the catalytic domain C of viral DNA polymerase [6]. The primary resistance mutations result in the replacement of the methionine by valine, isoleucine, or occasionally serine and are designated as rtM204V/I [4, 6]. These mutations (rtM204V/I) confer cross-resistance to lamivudine, telbivudine, and other members that belong to the L-nucleoside structural group such as emtricitabine and clevudine and reduce susceptibility to entecavir [4–8]. Moreover, it caused significant reduction in the antigenicity of immune-escaped HBsAg [9]. YMDD mutations are also an independent risk factor for HCC in liver cirrhosis patients [10]. YMDD mutations play an important role in the chronic hepatitis B management [4–6].

*Sophora alopecuroides *L. has been widely used for the treatment of liver disease in China. Oxymatrine (OMTR) (MW: 264.31) is one of the most pharmacologically active components in* Sophora alopecuroides *L. and had also been found to be capable of inhibiting HBV and relieving hepatic fibrosis [11–16]. It has been approved for the treatment of hepatitis B by the State Food and Drug Administration of China and is listed as one of the recommended anti-HBV drugs in the Guideline for Prevention and Treatment of CHB jointly proposed by the Chinese Society of Hepatology and the Chinese Society of Infectious Diseases. The rate of YMDD mutations is 24% after 1 year of lamivudine treatment [17]. Several reports revealed that combination of OMTR and lamivudine reduced the incidence of YMDD mutations caused by lamivudine. However, convincing evidence of lamivudine plus OMTR therapies is still needed. In addition, these studies, published in Chinese, cannot be accessed by non-Chinese speaking scientists.

The aim of this study was to evaluate the clinical value of oxymatrine in preventing lamivudine induced YMDD mutation using meta-analysis of data from published randomized controlled trials (RCTs) and to provide some useful information for clinical treatment and future research of YMDD mutation.

## 2. Materials and Methods

### 2.1. Eligibility Criteria

The inclusion criteria were the following: (i) Clinical diagnosis must meet the diagnostic criteria for CHB (Chinese Commission of Infectious and Parasitic Diseases, Viral Hepatitis Prevention and Treatment Programs); (ii) the included RCT studies were designed to compare the therapeutic effects of lamivudine therapy or lamivudine plus OMTR therapies in CHB patients; patients coinfected with other viral infections (HAV, HCV, HDV, and HEV) or hepatic cellular cancers were excluded; (iii) patients were treated for at least 48 weeks. Reports of duplicated studies were excluded by examining the author list, parent institution, sample size, and results.

### 2.2. Outcome Measure

The primary outcome was the incidence of YMDD mutation, and other measures included the end-of-treatment viral response (ETVR), alanine transaminase (ALT) normalization, HBeAg loss, HBeAg seroconversion, and occurrence of adverse events. The incidence of YMDD mutation was defined as detectable YMDD mutation by a sensitive test after treatment. ETVR was defined as undetectable HBV DNA at the end of treatment.

### 2.3. Information Sources and Searches

A search of the literature was conducted for studies that reported the therapeutic effects of lamivudine with or without OMTR therapies in CHB patients. The Cochrane Central Register of Controlled Trials, Medline, Science Citation Index, EMBASE, China National Knowledge Infrastructure, Wanfang Database, and China Biomedical Database were searched to identify RCTs published in the field of antiviral therapy for CHB. All the databases above were searched from their date of inception onwards until June 1, 2015, and irrespective of language or publication status. The keywords used in the literature searches included the following: chronic hepatitis B, hepatitis B virus, oxymatrine, lamivudine, YMDD, rtM204I, rtM204V, treatment, and trial.

### 2.4. Study Selection and Data Collection

Two authors (Min He and Yu Wu) independently screened titles and abstracts for potential eligibility and the full texts for final eligibility. We extracted the data from the included trials independently for quantitative analyses, and any disagreement was subsequently resolved by discussion.

### 2.5. Assessment of Risk of Bias in Included Studies

Two authors (Mengmeng Wang and Wenwen Chen) independently assessed the risk of bias for each included randomised trial. Disagreements were resolved by discussion with Jian Jiang and Weian Yuan. We assessed the following domains [18].

#### 2.5.1. Allocation Sequence Generation

- Low risk of bias: sequence generation was achieved using computer random number generation or a random number table. Drawing lots, tossing a coin, shuffling cards, and throwing dice are adequate if performed by an independent adjudicator.
- Uncertain risk of bias: the trial was described as randomised, but the method of sequence generation was not specified.
- High risk of bias: the sequence generation method is not, or may not be, random. Quasi-randomised studies, those using dates, names, or admittance numbers in order to allocate patients, are inadequate and will be excluded for the assessment of benefits but not for harm.

#### 2.5.2. Allocation Concealment

- Low risk of bias: allocation was controlled by a central and independent randomization unit, sequentially numbered, opaque, and sealed envelopes or similar, so that intervention allocations could not have been foreseen in advance of, or during, enrolment.
- Uncertain risk of bias: the trial was described as randomised but the method used to conceal the allocation was not described, so that intervention allocations may have been foreseen in advance of, or during, enrolment.
- High risk of bias: the allocation sequence was known to the investigators who assigned participants or the study was quasi-randomised. Quasi-randomised studies will be excluded for the assessment of benefits but not for harm.

#### 2.5.3. Blinding

- Low risk of bias: the trial was described as blinded, the parties were blinded, and the method of blinding was described, so that knowledge of allocation was adequately prevented during the trial.
- Uncertain risk of bias: the trial was described as blinded, but the method of blinding was not described, so that knowledge of allocation was possible during the trial.
- High risk of bias: the trial was not blinded, so that the allocation was known during the trial.

#### 2.5.4. Incomplete Outcome Data

- Low risk of bias: the numbers and reasons for dropouts and withdrawals in all intervention groups were described or it was specified that there were no dropouts or withdrawals.
- Uncertain risk of bias: the report gave the impression that there had been no dropouts or withdrawals, but this was not specifically stated.
- High risk of bias: the numbers or reasons for dropouts and withdrawals were not described.

#### 2.5.5. Selective Outcome Reporting

- Low risk of bias: predefined or clinically relevant and reasonably expected outcomes are reported on.
- Uncertain risk of bias: not all predefined or clinically relevant and reasonably expected outcomes are reported on or are not reported fully, or it is unclear whether data on these outcomes were recorded or not.
- High risk of bias: one or more clinically relevant and reasonably expected outcomes were not reported on; data on these outcomes were likely to have been recorded.

#### 2.5.6. Other Biases

- Low risk of bias: the trial appears to be free of other sources of bias (e.g., conflict of interests bias).
- Uncertain risk of bias: there is insufficient information to assesswhether other sources of bias are present.
- High risk of bias: it is likely that potential sources of bias related to specific design used, early termination due to some data-dependent process, lack of sample size or power calculation, or other bias risks are present.

Authors' judgements were based on the definitions of the above-listed domains, and trials with adequate assessments in all of the abovementioned bias risks domains were considered as having low risk of bias. Otherwise, a trial was considered with high risk of bias.

### 2.6. Assessment of Heterogeneity

We planned to use the Chi-squared statistic to assess heterogeneity and *I*-square statistic to measure inconsistency [19].

### 2.7. Assessment of Publication Biases

We planned to use the funnel plot to investigate publication biases if there were more than ten included trials.

### 2.8. Statistical Analysis

Meta-analysis was performed using fixed effect or random effect methods, depending on the absence or presence of significant heterogeneity. We used the relative risk (RR) of the main dichotomous outcomes as the measure of efficacy. The 95% confidence interval (CI) for the combined RR was also provided. The overall effect was tested using *z* scores calculated by Fisher's *z* transformation, with significance set at *p* < 0.05. Data analysis was carried out with the use of Review Manager Software 5.3.2 (Cochrane Collaboration, Oxford, United Kingdom).

## 3. Results

### 3.1. Literature Search

**Figure 1:**
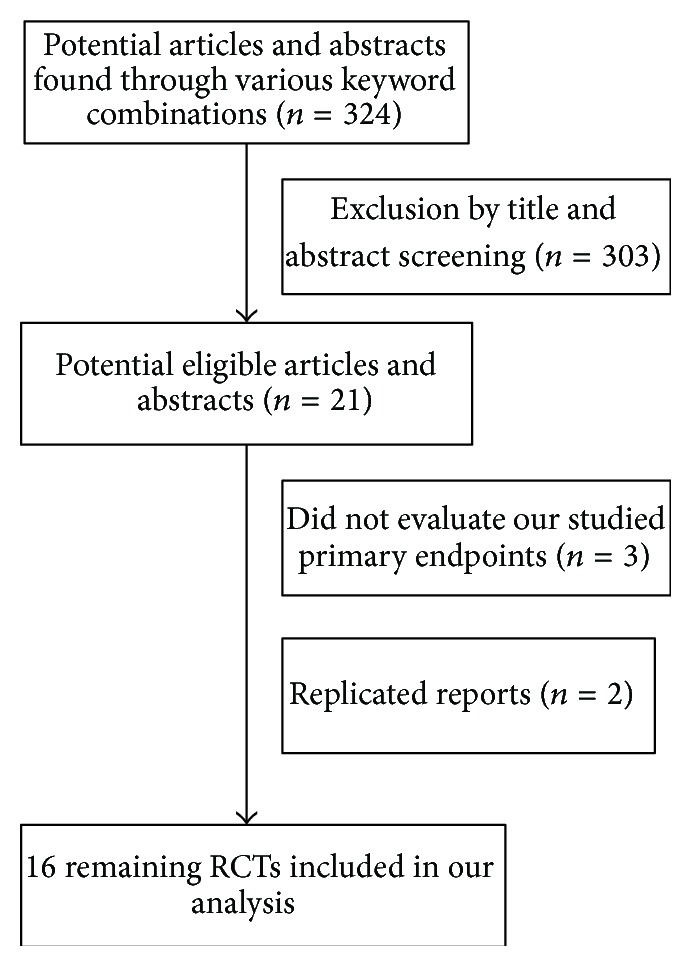
Analysis of the search results.

Figure 1 shows the results of the study screen. The literature search yielded 324 studies, 16 of which matched the selection criteria [20–35]. The combined CHB patient total was 1569.

### 3.2. Patient Characteristics and Study Quality

All RCTs included were published as full-length articles. The patients included in the sixteen trials were randomly assigned to accept lamivudine plus OMTR therapies or lamivudine therapy alone. Of the 1569 patients, 824 patients had therapy with lamivudine plus OMTR, and 745 patients had therapy with lamivudine alone. All studies were single-centre trials. The baseline characteristics of the sixteen included trials were summarized in Tables 1 and 2.

### 3.3. Risk of Bias in Included Studies

**Figure 2:**
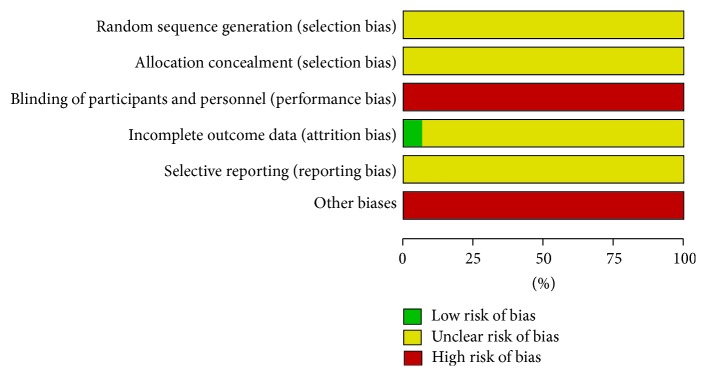
Risk of bias graph: reviewing authors' judgements about each risk of bias item presented as percentages across all included studies.

**Figure 3:**
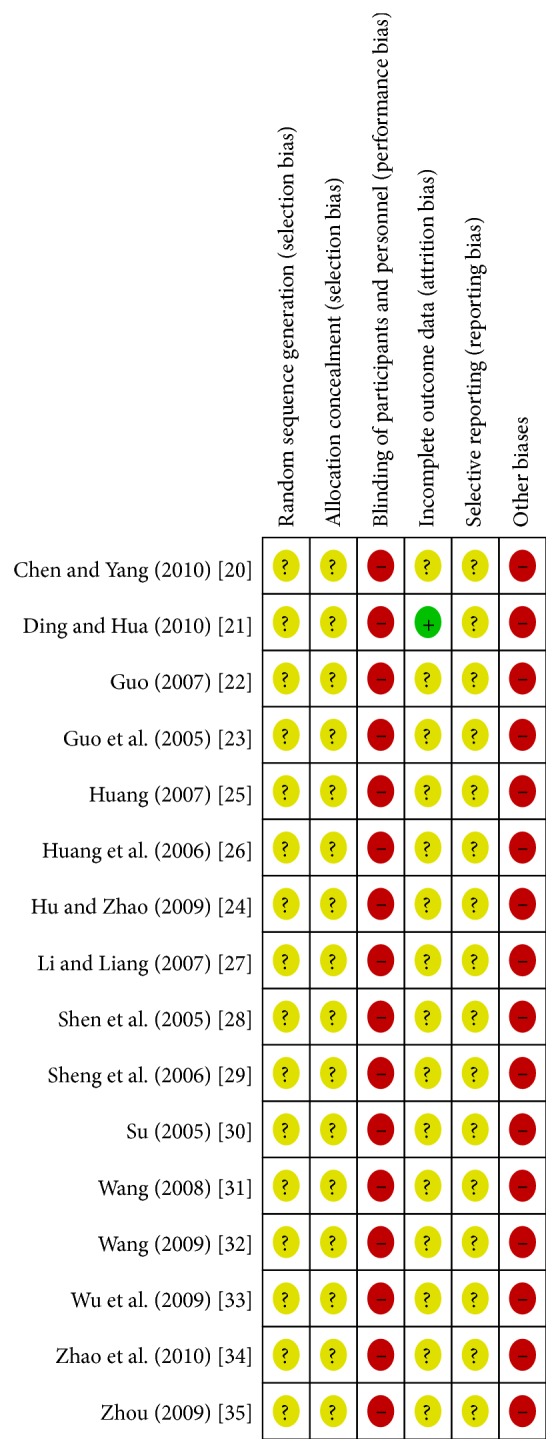
Risk of bias summary: reviewing authors' judgements about each risk of bias item for each included study.

The risk of bias of included trials is summarised in Figures 2 and 3. Following the risk of bias components, all trials included were classified as trials with high risk of bias.

### 3.4. Comparison of Lamivudine Plus OMTR Therapies and Lamivudine Therapies Alone

**Figure 4:**
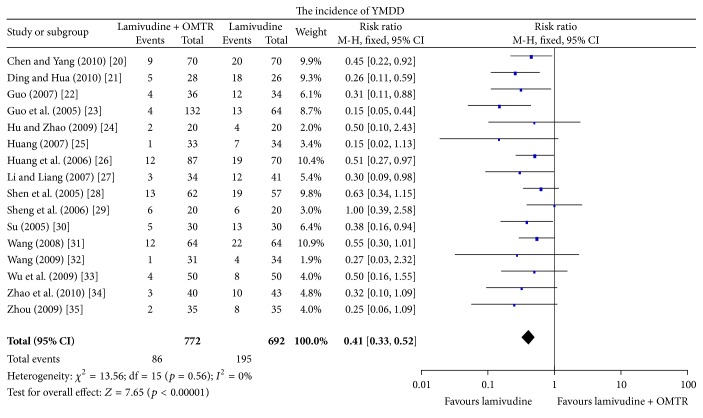
The incidence of YMDD mutation: comparison of lamivudine plus OMTR therapies and lamivudine therapy. RR, relative risk; CI, confidence interval; test for heterogeneity: Chi-squared statistic with its degrees of freedom (df) and *p* value; inconsistency among results: *I* ^2^ test for overall effect; *Z* statistic with *p* value.

**Figure 5:**
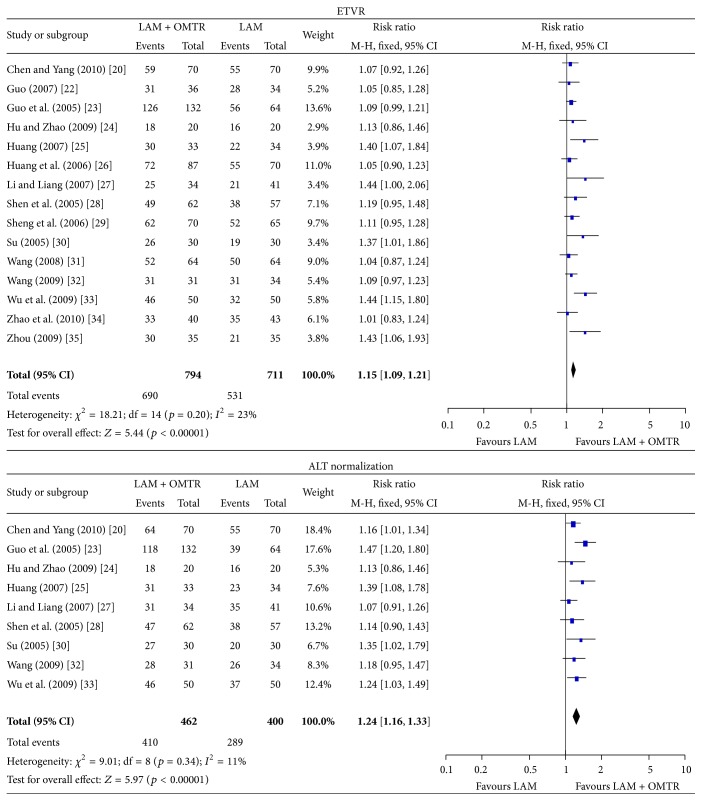
ETVR and ALT normalization: comparison of lamivudine plus OMTR therapies and lamivudine therapy. RR, relative risk; CI, confidence interval; test for heterogeneity: Chi-squared statistic with its degrees of freedom (df) and *p* value; inconsistency among results: *I* ^2^ test for overall effect; *Z* statistic with *p* value.

**Figure 6:**
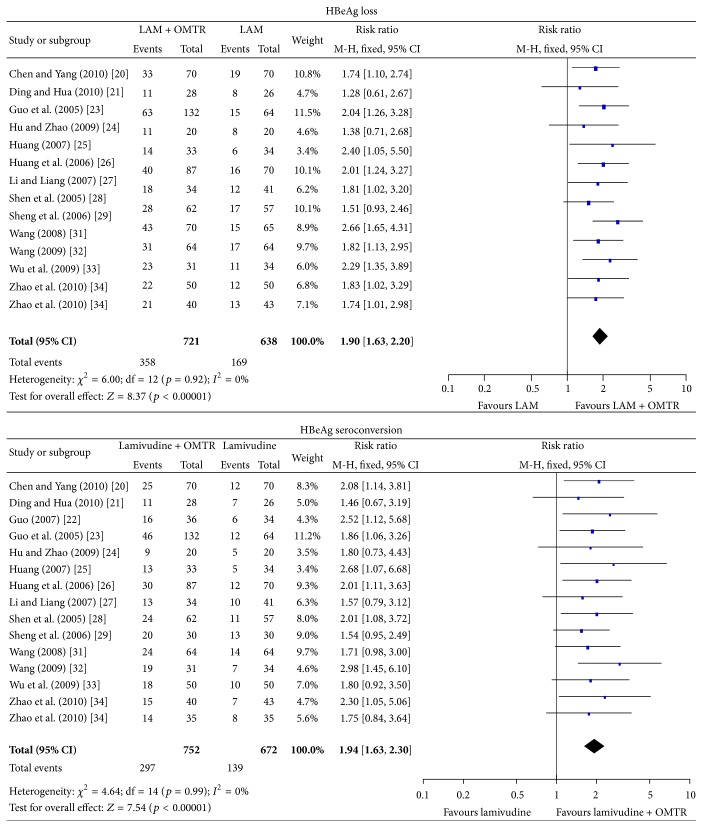
HBeAg loss and HBeAg seroconversion: comparison of lamivudine plus OMTR therapies and lamivudine therapy. RR, relative risk; CI, confidence interval; test for heterogeneity: Chi-squared statistic with its degrees of freedom (df) and *p* value; inconsistency among results: *I* ^2^ test for overall effect; *Z* statistic with *p* value.

In this study, the combined therapies of lamivudine plus OMTR were superior to lamivudine monotherapy. Patients treated with lamivudine plus OMTR achieved lower incidence of YMDD mutation than patients treated only with lamivudine 11.14% (86/772) versus 28.18% (195/692); RR: 0.41; 95% CI: 0.33–0.52; *p* < 0.05 (Figure 4). ETVRs were also higher in patients treated with combined therapies compared to the patients treated with lamivudine alone (86.90% (690/794) versus 74.68% (531/711); RR: 1.15; 95% CI: 1.09–1.21; *p* < 0.05) (Figure 5). Patients treated with combined therapies also achieved significantly higher ALT normalization, HBeAg loss, and HBeAg seroconversion (ALT normalization: 88.74% (410/462) versus 72.25% (289/400); RR: 1.24; 95% CI: 1.16–1.33; *p* < 0.05. HBeAg loss: 49.65% (358/721) versus 26.49% (169/638); RR: 1.90; 95% CI: 1.63–2.20; *p* < 0.05. HBeAg seroconversion: 39.49% (297/752) versus 20.68% (139/672); RR: 1.94; 95% CI: 1.63–2.30; *p* < 0.05) (Figures 5 and 6). In this meta-analysis for the incidence of YMDD mutation, ETVR, ALT normalization, HBeAg loss, and HBeAg seroconversion, there was no apparent heterogeneity.

### 3.5. Safety Profile Evaluation

Four included trials [21, 26, 29, 34] reported side effects. Adverse events were also reported in the included trials (including itch of skin, bellyache, diarrhoea, and fever). The overall adverse events showed no difference in patients treated with lamivudine plus OMTR and in patients treated with lamivudine alone, according to the reports of the included trials.

### 3.6. Publication Bias

We performed funnel plot analysis for the incidence of YMDD mutation to explore publication bias. All sixteen trials included for a funnel plot analysis of lamivudine plus OMTR therapies versus lamivudine therapy lay within the 95% CI line. These results implied the existence of some publication bias.

## 4. Discussion

As new generations of anti-HBV drugs are available, the application of lamivudine was reducing; thus less correlation studies were reported. However, lamivudine is still used in some low-income countries due to the high cost of new antiviral drugs [2]. Lamivudine is an inexpensive agent, but it engenders very high rates of resistance with long-term monotherapy. The resistance generation is closely associated with mutations in the highly conserved YMDD motif [6]. Lamivudine therapy would increase the risk of YMDD mutations 5.23 times higher than the untreated patients. It is notable that YMDD-motif mutation can also occur naturally with a rather high rate about 12.21% among untreated CHB patients [36]. The prevalence of drug-resistant mutants in patients is associated with the loss of clinical and virological benefits and may limit future therapeutic options. So prevention is important for long-term therapeutic efficacy. In successful antiviral therapy of patients, drug combinations can delay or prevent the emergence of drug-resistant mutants.

In this study, we have summarized the available evidence from RCTs comparing lamivudine monotherapy with lamivudine plus OMTR therapies for the treatment of CHB. Our results suggest that combination therapies of lamivudine plus OMTR may achieve significantly lower incidence of YMDD mutations than lamivudine monotherapy. Combination therapies of LAM plus OMTR have also shown superior ETVRs, ALT normalizations, HBeAg loss, and HBeAg seroconversion.

Ideally, drugs used in combination should have different mechanisms of action and act additively or synergistically. Current oral treatments for CHB are all nucleoside/nucleotide reverse transcriptase inhibitors that inhibit HBV DNA replication by targeting the HBV DNA polymerase. OMTR acts by multiple mechanisms that involve both antiviral and immunomodulatory effects. These include direct antiviral effect, activation of host antiviral enzymes, and stimulation of cellular immune responses against HBV-infected hepatocytes [11–16, 37, 38]. The efficacy of OMTR is bound up with its multiple pharmacological activities. It should be mentioned here that the anti-HBV effect of OMTR is mediated through heat-stress cognate 70 (Hsc70) downregulations (an indirect effect). Hsc70 is a host protein which supports HBV DNA replication [39]. OMTR significantly suppressed HBV de novo synthesis at the reverse transcription stage from pgRNA to DNA and was active against either wild-type HBV or variants resistant to lamivudine, adefovir, and entecavir [39, 40]. The anti-HBV effect of OMTR was mediated through destabilizing Hsc70 mRNA; Hsc70 mRNA 3′UTR sequence was the element responsible for the destabilization effect of OMTR [39]. These findings suggested that OMTR therapies are associated with lower incidence of YMDD mutations and are more efficacious in the treatment of CHB patients when combined with lamivudine.

But it must be noted that this meta-analysis had some limitations. Firstly, the quality of the methodological design of individual studies was not high. Secondly, the asymmetric funnel plot implied that publication biases may occur. Thirdly, the diversity of treatment dose and the small sample number and the lack of long-term follow-ups degraded the validity of the evidence of the clinical trials.

## 5. Conclusions

Combined therapies of lamivudine plus OMTR yielded a lower incidence of YMDD mutation than lamivudine monotherapy. This finding provided some useful information for clinical treatment and future research of YMDD mutation. Considering that this meta-analysis had the limitations in some ways, the exact outcome needs to perform rigorously designed, multicenter, and large randomized controlled trials.

## Figures and Tables

**Table 1:** Characteristics of the trials included in the meta-analysis.

References	Sample size (treatment/control)	Gender (male/female)	Age	Treatment duration (weeks)	YMDD mutation (pretreatment) (treatment/control)
Chen and Yang (2010) [20]	70/70	82/58	19∼61	52	0/0
Ding and Hua (2010) [21]	30/34^a^	41/23	18∼49	260	0/0
Guo (2007) [22]	36/34	60/10	38.5	52	0/0
Guo et al. (2005) [23]	132/64	—/—	—	52	0/0
Hu and Zhao (2009) [24]	20/20	27/13	31.25	52	0/0
Huang (2007) [25]	33/34	52/15	19∼55	56	0/0
Huang et al. (2006) [26]	87/70	95/62	39.1	52	0/0
Li and Liang (2007) [27]	34/41	54/21	18∼58	52	0/0
Shen et al. (2005) [28]	62/57	—/—	18∼58	104	0/0
Sheng et al. (2006) [29]	70/65^a^	109/26	18∼65	52∼104	0/0
Su (2005) [30]	30/30	—/—	34.89 ± 11.13	52	0/0
Wang (2008) [31]	64/64	77/51	18∼54	52	0/0
Wang (2009) [32]	31/34	38/27	50.1	48	0/0
Wu et al. (2009) [33]	50/50	54/46	22∼62	48	0/0
Zhao et al. (2010) [34]	40/43	52/31	16∼62	78	0/0
Zhou (2009) [35]	35/35	55/15	19∼61	52	0/0

^a^Patients did not complete detection of YMDD mutation and data is shown.

**Table 2:** Interventions of the trials included in the meta-analysis.

References	Intervention	
	Treatment (OMTR plus lamivudine)	Control (lamivudine)
Chen and Yang (2010) [20]	Lamivudine (100 mg once-daily), OMTR capsules (200 mg thrice-daily)	lamivudine (100 mg once-daily)
Ding and Hua (2010) [21]	Lamivudine (100 mg once-daily), OMTR capsules (200 mg thrice-daily)	lamivudine (100 mg once-daily)
Guo (2007) [22]	Lamivudine (100 mg once-daily), OMTR (beginning 4 weeks, OMTR injection 600 mg/day; the remaining weeks, OMTR capsules 200 mg thrice-daily)	lamivudine (100 mg once-daily)
Guo et al. (2005) [23]	Lamivudine (100 mg once-daily), OMTR (beginning 8 weeks, OMTR injection 600 mg/day; the remaining 26 weeks, OMTR capsules 200 mg thrice-daily)	lamivudine (100 mg once-daily)
Hu and Zhao (2009) [24]	Lamivudine (100 mg once-daily), OMTR capsules (200 mg thrice-daily)	lamivudine (100 mg once-daily)
Huang (2007) [25]	Lamivudine (100 mg once-daily), OMTR capsules (beginning 28 weeks, 200 mg thrice-daily)	lamivudine (100 mg once-daily)
Huang et al. (2006) [26]	Lamivudine (100 mg once-daily), OMTR capsules (beginning 26 weeks, 200 mg thrice-daily)	lamivudine (100 mg once-daily)
Li and Liang (2007) [27]	Lamivudine (100 mg once-daily), OMTR capsules (200 mg thrice-daily)	lamivudine (100 mg once-daily)
Shen et al. (2005) [28]	Lamivudine (100 mg once-daily), OMTR (beginning 8 weeks, OMTR injection 600 mg/day; the remaining 18 weeks, OMTR capsules 200 mg thrice-daily)	lamivudine (100 mg once-daily)
Sheng et al. (2006) [29]	Lamivudine (100 mg once-daily), OMTR (at 26 weeks after lamivudine treatment, OMTR injection 600 mg/day, 8 weeks)	lamivudine (100 mg once-daily)
Su (2005) [30]	Lamivudine (100 mg once-daily), OMTR (beginning 13 weeks, OMTR injection 600 mg/day; the remaining weeks, OMTR capsules 100 mg thrice-daily)	lamivudine (100 mg once-daily)
Wang (2008) [31]	Lamivudine (100 mg once-daily), OMTR capsules (beginning 26 weeks, 200 mg thrice-daily)	lamivudine (100 mg once-daily)
Wang (2009) [32]	Lamivudine (100 mg once-daily), OMTR capsules (200 mg thrice-daily)	lamivudine (100 mg once-daily)
Wu et al. (2009) [33]	Lamivudine (100 mg once-daily), OMTR capsules (beginning 24 weeks, 200 mg thrice-daily)	lamivudine (100 mg once-daily)
Zhao et al. (2010) [34]	Lamivudine (100 mg once-daily), OMTR capsules (beginning 26 weeks, 200 mg thrice-daily)	lamivudine (100 mg once-daily)
Zhou (2009) [35]	Lamivudine (100 mg once-daily), OMTR (beginning 13 weeks, OMTR injection 600 mg/day; the remaining weeks, OMTR capsules 200 mg thrice-daily)	lamivudine (100 mg once-daily)

OMTR: oxymatrine.
